# Optimizing Collagen Biostimulator Choice in LATAM: Expert Consensus on Patient Selection, Ethnic Skin Phenotypes, and Accessibility

**DOI:** 10.1111/jocd.70564

**Published:** 2025-11-28

**Authors:** Eugenia Cure, Luis Alberto Parra Hernández, Andreina Martinez Amado, Juan Sebastian Rodriguez Cabrales, Eliana Garcés, Valentina Dicker, Desiree Castelanich, Carolina Schneider, Ingrid Salas, Joselyn Argueta, Lina Velázquez Tafur, Andrea Acevedo, Alejandra Bugallo, Evalicia Murúa, Andrea Marcela Parra Hernandez

**Affiliations:** ^1^ University J. N. Corpas Barranquilla Colombia; ^2^ Aesthetic Medicine, Sociedad Internacional de Rejuvenecimiento facial no Quirúrgico (SIRF) North University Barranquilla Colombia; ^3^ Aesthetic Medicine, Clinical Research University of Salamanca Bogota Colombia; ^4^ Aesthetic Medicine, Clinical Research National University of Colombia Bogotá Colombia; ^5^ Plastic and Aesthetic Surgery Military University of Colombia Barranquilla Colombia; ^6^ Private Practice, Regenera Skin Barranquilla Colombia; ^7^ Aesthetic Medicine University of Rosario Bogotá Colombia; ^8^ Sociedad Argentina de Dermatología University of Buenos Aires Buenos Aires Argentina; ^9^ Plastic and Aesthetic Surgery University of Buenos Aires Buenos Aires Argentina; ^10^ Private Practice, Dermatosphera Skin Clinic Cartagena Colombia; ^11^ Private Practice, Rejuvé Aesthetic Clinic Guatemala City Guatemala; ^12^ Universidad of Valle Cali Colombia; ^13^ Aesthetic Medicine Universidad del Rosario Cali Colombia; ^14^ University of Buenos Aires Buenos Aires Argentina; ^15^ Private Practice, Beauty Care Clinic Buenos Aires Argentina; ^16^ Private Practice, Dra Evalicia Murúa Clinic Mexico City Mexico; ^17^ Sociedad Internacional de Rejuvenecimiento facial no Quirúrgico (SIRF) Barranquilla Colombia

**Keywords:** calcium hydroxylapatite, collagen biostimulators, LATAM consensus, poly‐l‐lactic acid, regenerative aesthetics

## Abstract

**Background:**

The diverse ethnic skin phenotypes and socioeconomic landscape of Latin America (LATAM) necessitate tailored approaches to collagen biostimulators, calcium hydroxylapatite (CaHA) and poly‐l‐lactic acid (PLLA). A region‐specific consensus on their optimal use is lacking.

**Objectives:**

To establish expert consensus guidelines on patient selection, techniques, and practical application of CaHA and PLLA for collagen biostimulation in LATAM populations.

**Methods:**

A modified Delphi process was conducted with 14 LATAM experts. Following a systematic literature review, 58 statements were drafted across key clinical domains. Consensus was defined as ≥ 75% agreement, stratified as strong (≥ 85%) or moderate (75%–84%).

**Results:**

Strong consensus was achieved on 51 statements (87.9%), with 4 reaching moderate consensus. Key recommendations include: selecting CaHA for thicker skin and immediate volumization, and PLLA for thinner skin and long‐term collagen remodeling; using specific dilution protocols (e.g., CaHA 1:1 for face, 1:4 for body); employing cannulas for safety; and implementing vigorous post‐PLLA massage. Initial disagreements on age‐based restrictions were resolved through literature review, achieving final consensus.

**Conclusion:**

This consensus provides the first LATAM‐specific framework for CaHA and PLLA use, integrating ethnic diversity, anatomical considerations, and accessibility to optimize safety and efficacy in regenerative aesthetics.

## 1 Introduction

The escalating demand for non‐surgical aesthetic procedures has driven significant advancements in injectable biostimulators, which aim to rejuvenate aging skin by stimulating endogenous collagen production [1]. Collagen biostimulators such as calcium hydroxylapatite (CaHA) and poly‐l‐lactic acid (PLLA) address age‐related collagen depletion and extracellular matrix (ECM) degradation by acting as regenerative scaffolds or bioactive cues to promote natural collagen synthesis [2].

LATAM populations exhibit ethnically diverse skin phenotypes (e.g., mestizo, indigenous, afro‐descendant Fitzpatrick IV–VI) with documented variations in collagen architecture (e.g., fibril density, elastin fragmentation) and fibroblast behavior (e.g., TGF‐β1 sensitivity, MMP‐1 expression) [3, 4]. These differences arise from genetic polymorphisms (COL1A1 rs1800012, COL3A1 haploinsufficiency) and epigenetic factors (UV‐induced hypermethylation of TIMP promoters) that modulate inflammatory responses to biostimulators [3, 5, 6]. This consensus integrates these phenotypes into clinical protocols.

Calcium hydroxylapatite (CaHA), the primary component of the dermal filler Radiesse, functions as a collagen biostimulator through a dual mechanism combining immediate volumization and long‐term neocollagenesis. Its bioceramic microspheres (25–45 μm) interact with fibroblasts and the ECM, restoring mechanical tension via mechanotransduction—a process that directly activates fibroblasts to synthesize collagen I, III, and elastin [7, 8]. This scaffold‐like activity promotes tissue regeneration with minimal inflammatory involvement, distinguishing CaHA from traditional inflammatory‐dependent agents [7].

In contrast, poly‐l‐lactic acid (PLLA) stimulates collagen production through a controlled foreign body response. When injected, irregularly shaped PLLA microparticles recruit monocytes and macrophages, which fuse into multinucleated giant cells. This immune cascade upregulates growth factors such as TGF‐β1 and tissue inhibitors of metalloproteinases (TIMPs), inducing fibroblast‐mediated collagen synthesis over weeks to months [9, 10]. When injected, PLLA microparticles (size 2 to 150 μm) [11] are recognized as foreign bodies, triggering monocytes to recruit macrophages, which fuse into multinucleated giant cells [9]. This process upregulates growth factors like TGF‐β1 and tissue inhibitors of metalloproteinases (TIMPs), promoting fibroblast activation and the synthesis of type I and III collagen [12]. Unlike immediate fillers, PLLA's effects are gradual, with results emerging over weeks to months as the particles degrade, leaving behind a neocollagenous matrix that improves skin thickness, elasticity, and volume [12].

While current literature offers robust recommendations for the standalone use of CaHA and PLLA [10, 13, 14, 15], there is limited evidence guiding clinicians on when to prioritize one biostimulator over the other in specific clinical scenarios. This gap is particularly significant in Latin America (LATAM), where diverse ethnicities, skin types, and aging phenotypes demand tailored approaches to optimize efficacy and minimize adverse events.

The LATAM Aesthetic Consensus Group presents the first region‐specific guidelines to address this unmet need, integrating mechanistic insights with practical expertise. This consensus establishes criteria for patient selection, combination strategies with adjunct therapies, and protocols addressing ethnic variations in collagen turnover and tissue response. By bridging mechanistic knowledge with clinical application, this work aims to enhance decision‐making precision and improve outcomes in aesthetic practice across LATAM populations.

## 2 Methods

A panel of 14 experts was selected from Colombia, Argentina and Guatemala, experts in the fields of Plastic Surgery, Dermatology, Ophthalmology, Oculoplastic Surgery and Aesthetic Medicine, a number consistent with common Delphi methodology to balance diversity of opinion with group manageability. Selection was based on clinical expertise, publication record, and geographic practice within LATAM to capture a range of perspectives, though we acknowledge that representation from all sub‐regions was not achieved.

This study utilized a modified Delphi method to develop clinical consensus guidelines. According to the International Committee of Medical Journal Editors (ICMJE) guidelines and common institutional policies, this type of expert consensus development, which does not involve patient data or interventions, is often considered exempt from formal ethical review board approval. Nevertheless, the study was conducted in accordance with the principles of the Declaration of Helsinki. All participating experts provided formal written consent for their anonymous responses to be collected, analyzed, and published prior to their enrollment in the study.

The experts answered a series of questionnaires between January and March 2025 to give recommendations regarding the use of biostimulators (Calcium Hydroxylapatite and Polylactic Acid). Experts were selected to be part of the panel based on their medical expertise, experience and familiarity with the use of CaHa and PLLA (mean duration of 5 years of experience with these products).

Inclusion criteria comprised a structured literature review performed on PubMed to inform the initial drafting of statements on the use of Calcium Hydroxylapatite (CaHA) and Poly‐l‐Lactic Acid (PLLA) as biostimulators for facial rejuvenation and collagen stimulation. The search included articles published in English between January 2019 and January 2025, using the following terms: “Calcium Hydroxylapatite,” “Poly‐l‐Lactic Acid,” “biostimulators,” “collagen stimulation,” “facial rejuvenation,” “injection techniques,” “dilution protocols,” “complications,” and “long‐term outcomes.”

**FIGURE 1:**
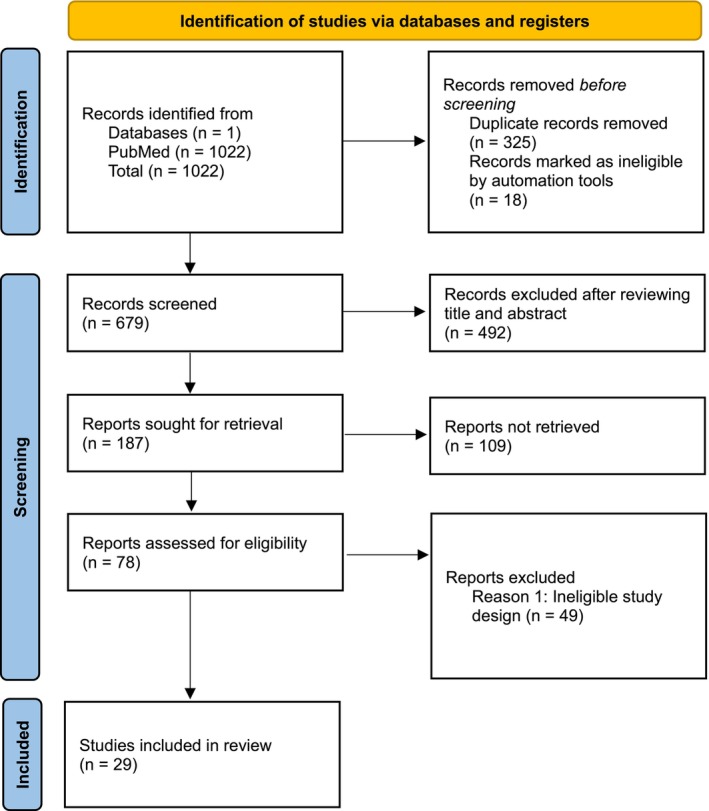
PRISMA 2020 flow diagram.

Studies were prioritized based on the highest level of evidence, with international guidelines and expert opinions considered first, followed by randomized controlled trials (RCTs), observational studies, and case series. The search focused on human studies that addressed key aspects of biostimulator use, including patient selection criteria, injection techniques, dilution protocols, expected outcomes, and management of complications. The panel also reviewed references cited in the selected articles to identify additional relevant publications. Special attention was given to studies that provided insights into regional practices in Latin America, socioeconomic factors influencing biostimulator selection, and patient expectations. Exclusion criteria were non‐human studies, articles not published in English, and studies that did not specifically address injection techniques, dilution protocols, safety outcomes, or long‐term efficacy. A PRISMA flow diagram has now been included as Figure 1 to illustrate in detail the results of our article selection process and to provide a transparent overview of the number of records identified, screened, excluded, and finally included in the review.

Findings from the literature review were synthesized by the steering committee and thematically mapped to key clinical domains (personal election of biostimulators, the socioeconomic factors influencing the selection criteria in the region, exclusion criteria on biostimulators, patient indications, including age, corporal regions and profiles, injection techniques, dilution protocols, the expected results after the treatment, including patients' expectations, recommendations after the treatment, complications and factors influencing success or failure for long‐term treatment). These evidence‐based themes were used to draft the initial 58 Delphi statements, ensuring the process was grounded in the current literature.

The literature search yielded 29 studies that met the inclusion criteria. These studies were deemed to provide the most direct and influential evidence relevant to the objectives of this consensus. These key studies were synthesized by the steering committee to inform the drafting of the Delphi statements. Their authors, study types, and primary findings relevant to biostimulator use in LATAM are summarized in Table 1.

**TABLE 1:** Key evidence from the systematic literature review informing the LATAM consensus statements on CaHA and PLLA.

Citation	Title	Study type	Bio stimulator	Key findings	Consensus domain
Alcotzer et al. (2023)	Optimal Changes Seen in Patients After Treatment With Poly‐l‐Lactic Acid—Retrospective Descriptive Study	Retrospective Descriptive Study	PLLA	Repeated PLLA injections produced progressive, durable lateral midface and lower‐third volumization and contour enhancement; majority of evaluator ratings were moderate–strong Reported antiptotic (lifting) effects with secondary improvement in nasolabial folds and marionette lines; enhanced jawline definition and masseter contour noted Skin quality improvements (texture, firmness, pore size, radiance, hydration) with objective 3D surface imaging evidence and histologic support for neocollagenesis Typical course: multiple sessions (commonly 2–3); optimal responders received higher mean vials (~3.8 vials); effects emerge over months and may peak ~6–12 months Safety: mostly mild transient events (bruising, swelling, small nodules) with nodules generally resolving without intervention	Expected Outcomes; Safety Protocols
Amiri et al. (2024)	A Systematic Review and Meta‐analysis of Single‐group Studies Assessing the Role of Calcium Hydroxylapatite in Aesthetic Enhancements and Satisfaction	Systematic Review	CaHA	Pooled single‐group studies (*n* = 46) show > 90% patient satisfaction and investigator‐reported improvement on global aesthetic scales Most included studies were single‐group designs; highlights high real‐world satisfaction but limited high‐quality comparative evidence	Expected Outcomes; Patient Selection
Bohnert et al. (2019)	Randomized, Controlled, Multicentered, Double‐Blind Investigation of Injectable Poly‐l‐Lactic Acid for Improving Skin Quality	Randomized Controlled Trial	PLLA	PLLA group showed significant increases in skin hydration and elasticity at 12 months vs. control (hydration *p* < 0.0001; elasticity *p* = 0.0017) No treatment‐related adverse events reported; mild transient swelling resolved without sequelae	Expected Outcomes; Safety Protocols; Product Mechanisms
Bravo et al. (2020)	Safety in Immediate Reconstitution of Poly‐l‐Lactic Acid for Facial Biostimulation Treatment	Case Series	PLLA	Immediate reconstitution to 12 mL (5 mL SWFI + 2 mL 2% lidocaine etc.) with thorough mixing then injection via 22G cannula in fan retrograde technique; 58 facial applications, mean age 52.6 Nodule incidence 3.44% (2/58) within first month; resolved after intervention following dilution Post‐treatment massage 5‐5‐5 rule; follow‐up within 30 days for AEs	Dilution Protocols; Injection Techniques; Complication Management
Bravo et al. (2024)	Exploring the Safety and Satisfaction of Patients Injected With Collagen Biostimulators—A Prospective Investigation Into Injectable Poly‐l‐Lactic Acid (PLLA)	Prospective Cohort Study	PLLA	Patient‐reported immediate symptoms: pain 77.8%, redness 22.2%; clinically only one minor nodulation recorded (resolved) Highlights discrepancy between patient diary reporting and clinical exam—overreporting possible due to heightened awareness	Patient Selection; Safety Protocols; Complication Management
Christen et al. (2022)	Collagen Stimulators in Body Applications: A Review Focused on Poly‐l‐Lactic Acid (PLLA)	Narrative Review	PLLA	Reconstitution volumes evolved from 3 to 5 mL to up to 24 mL; higher reconstitution volumes (e.g., 7–9 mL with lidocaine) associated with reduced nodule rates Injection devices: needles 25–27G or cannulas (22G); techniques include retrograde linear threading, fanning; sessions typically 1–4, spaced 4–8 weeks Safety: nodules more common with low reconstitution volume (3–4 mL); hydration time 48–72 h historically recommended but immediate injection with high volume shown safe in recent studies	Dilution Protocols; Injection Techniques; Safety Protocols
Corduff et al. (2021)	Pan‐Asian Consensus on Calcium Hydroxyapatite for Skin Biostimulation, Contouring, and Combination Treatments	Consensus Guideline	CaHA	Most experts dilute CaHA 1:1 for biostimulation in Asian patients; hyperdilution (≥ 1:2) used for thinner skin or broader surface‐area tightening Prefer cannula delivery (commonly 25G; some use 22G) for subdermal/subcutaneous placement; avoid direct infraorbital injections for safety Dilution defined separately from dose; hand surface area ≈ one syringe (1.5 mL CaHA) as a practical dosing guide; use 1–1.5 cc per 100 cm^2^ for some body areas (e.g., abdomen) and 1–1.5 cc per hand for hand rejuvenation Injection planes: diluted CaHA for subdermal whole‐face/neck biostimulation; interfascial for temple contouring; supraperiosteal/subdermal for contouring when undiluted Combination treatments: perform MFU‐V before CaHA if same‐session treatments planned Practical safety/technique consensus: minimize entry ports, favor cannula, tailor dilution to skin thickness/region, and feather dilution (use less diluted near problematic areas)	Dilution Protocols; Injection Techniques; Safety Protocols; Patient Selection
de Almeida et al. (2023)	Efficacy and Tolerability of Hyperdiluted Calcium Hydroxylapatite (Radiesse) for Neck Rejuvenation: Clinical and Ultrasonographic Assessment	Prospective Cohort Study	CaHA	Dermal thickness, elasticity, and collagen/elastin markers increased at 4 and 7 months after hyperdilute CaHA (dilutions used: 1:2 for normal skin, 1:4 for thin skin, 1:6 for atrophic skin) Injection‐related AEs limited to transient bruising and swelling; tolerable Typically one syringe per session for neck; retrograde linear subdermal injections used; dilution tailored to skin thickness	Expected Outcomes; Dilution Protocols; Injection Techniques; Safety Protocols
Fakih et al. (2021)	Combining Calcium Hydroxylapatite and Hyaluronic Acid Fillers for Aesthetic Indications: Efficacy of an Innovative Hybrid Filler	Retrospective chart review (case series)	CaHA	Premixed CaHA:CPM‐HA produced volumizing and lifting effects in cheeks and jawline with significant clinician‐rated improvement in jawline scores at 3 months (mean CR‐MASJ from 2.12 to 0.68) and sustained benefit at 12 months 100% of subjects had ≥ 1‐point CR‐MASJ improvement at 3 months; 85% at 12 months Mixing technique and ratios described (various CaHA:CPM‐HA ratios; subcutaneous fanning with 25G cannula) and practical protocol provided No adverse events reported in this cohort; limitations include retrospective design, small sample, variable mixing ratios, and lack of patient‐reported and 3D objective measures	Expected Outcomes; Injection Techniques; Safety Protocols
Fisher et al. (2024)	The Emerging Role of Biostimulators as an Adjunct in Facial Rejuvenation: A Systematic Review	Systematic Review	CaHA & PLLA	PLLA: evidence of neocollagenesis with clinical improvements; included 1 RCT among PLLA studies CaHA: multiple prospective studies (*n* = 242 total) demonstrating collagen deposition/skin thickness improvements Emphasizes gradual onset of effects—counsel patients to avoid expectation of immediate final results	Expected Outcomes; Patient Selection; Product Mechanisms
Galadari et al. (2024)	A Systematic Review of Radiesse (Calcium Hydroxylapatite): Evidence and Recommendations for the Body	Narrative Review	CaHA	Techniques for body: bolus/tenting, proximal‐to‐distal fanning with cannula; cannula associated with fewer AEs (3% vs. 24% with needle in one report) Studies report improvements in hand and body skin quality with 1:1 or hyperdilute CaHA; local/self‐limiting AEs predominated Recommends attention to correct plane (superficial lamina < 1 mm) to avoid visibility and complications	Injection Techniques; Safety Protocols; Expected Outcomes
Goldie et al. (2024)	Consensus Agreements on Regenerative Aesthetics: A Focus on Regenerative Biostimulation With Calcium Hydroxylapatite	Consensus Guideline	CaHA	Defined regenerative aesthetics (RA) distinct from regenerative medicine; RA aims to restore youthful tissue architecture/function. CaHA (Radiesse) chosen as exemplar: evidence for increased collagen I/III ratio, elastin, proteoglycans, dermal thickness, pliability, angiogenesis, and fibroblast activation supporting regenerative capacity Recommended multimodal assessment of regeneration including imaging, histology, and molecular benchmarks	Product Mechanisms; Expected Outcomes
Guida et al. (2023)	A Systematic Review of Radiesse (Calcium Hydroxylapatite) and Carboxymethylcellulose: Evidence and Recommendations for Treatment of the Face	Systematic Review	CaHA	CaHA/CMC proven safe and effective for cheeks, jawline, HIV‐related facial lipoatrophy, and nasolabial folds; supported by multiple RCTs and prospective studies Diluted (1:1) and hyperdiluted (≥ 1:2) preparations used for biostimulation/skin tightening; global expert consensus supports off‐label diluted use though further RCTs are needed Evidence summary: 5 RCTs, 10 prospective, 4 retrospective studies; favorable safety profile with mostly mild AEs; recommendations include cannula use and careful technique to minimize complications	Expected Outcomes; Dilution Protocols; Safety Protocols; Patient Selection
Haddad et al. (2022)	Evaluation of the Biostimulatory Effects and the Level of Neocollagenesis of Dermal Fillers: A Review	Narrative Review	CaHA & PLLA	All reviewed biostimulators (PLLA, CaHA, PCL, HA) show evidence of inducing neocollagenesis, neoelastinogenesis, and neovascularization, but the amount of collagen produced remains poorly quantified PCL and CaHA show persistent particle‐associated collagen deposition and longer‐lasting effects; CaHA associated with collagen‐III near particles that progresses to collagen‐I in an “onion” pattern in biopsies HA can induce procollagen and related pathway activation via mechanical stretch from cross‐linked HA (e.g., increased procollagen at 1–3 months) Animal and small human studies show variable inflammatory responses across products (e.g., eosinophils reported with some PCL studies; CaHA less eosinophilic), highlighting model‐dependent differences Overall conclusion: biostimulatory capacity is established but comparative quantitative data are limited; larger, well‐designed clinical trials with standardized histologic/molecular measures are needed	Product Mechanisms; Expected Outcomes
Haddad et al. (2025)	Injectable Poly‐l‐Lactic Acid for Body Aesthetic Treatments: An International Consensus on Evidence Assessment and Practical Recommendations	Expert consensus/Review	PLLA	Evidence supports PLLA‐SCA for non‐facial (body) aesthetic indications including neck, décolletage, buttocks, knees, abdomen, hands and upper arms with potential sustained aesthetic improvements (sagging, wrinkling, dimpling, cellulite) and minimal side effects reported Current evidence base is mainly prospective observational studies and case series; standardized injection protocols and validated clinical evaluation tools are needed for different body areas Mechanism: PLLA induces neocollagenesis and structural remodeling; differences among PLLA formulations may affect safety and performance, but head‐to‐head comparisons are lacking Ongoing and recent studies: trials and retrospective reviews evaluating PLLA‐SCA for arm laxity, décolletage wrinkles, hip dimple profile, cellulite, and multicenter safety for non‐facial use are in progress or reported Recommendations: expert‐led, evidence‐based guidance provided for injection protocols by body site and evaluation tools; international panel selected studies by treatment area to inform practical recommendations	Injection Techniques; Expected Outcomes
Hernandez et al. (2024)	Multilayer Technique Using Calcium Hydroxylapatite Biostimulation With Different Dilutions in the Lateral Face	Case Series/Technique Study	CaHA	Multilayer approach: supraperiosteal boluses for focal biostimulation (e.g., 0.15/0.15/0.1 mL) then subdermal 1:1 diluted CaHA for global biostimulation produced lateral‐face lift and regional repositioning effects Lateral‐first injections yield soft‐tissue repositioning, reducing filler volume required; safety: supraperiosteal plane favored to avoid vasculature Use of 27G needle for boluses (or cannula if preferred); diluted CaHA (1:1) placed in subdermal plane for biostimulation	Injection Techniques; Expected Outcomes; Safety Protocols; Dilution Protocols
Lin et al. (2024)	The AestheCode System: A Safe and Efficient Guide for AestheFill Injection	Narrative Review/Manual	CaHA	Recommends thin/super‐thin suspensions for most facial treatments to reduce nodule risk; repeat treatments > 1 month apart for larger deficits Provides site‐specific layer and tool guidance (e.g., subcutaneous/subdermal for most facial parts) and lower cohesivity for easier even injection and lower nodules	Injection Techniques; Complication Management; Safety Protocols
Lorenc et al. (2021)	Skin Tightening With Hyperdilute CaHA: Dilution Practices and Practical Guidance for Clinical Practice	Consensus Guideline/Practical Guidance	CaHA	Hyperdilute CaHA (≥ 1:2) provides biostimulation without volumization; 1:1 retains some volumization Recommended cannula use (22–25G) to minimize entry ports and evenly distribute product; minimize number of entry ports Dilution guidance by skin thickness: 1:2–1:3 for face, 1:2–1:3 neck/décolletage, 1:3 most body; 1:4 or more for thin/atrophic skin; lidocaine dosing caution (max 3–7 mg/kg)	Dilution Protocols; Injection Techniques; Safety Protocols; Patient Selection
Lorenc et al. (2021)	Consensus recommendations for CaHA use in Asian patients (excerpts)	Consensus Guideline	CaHA	CaHA commonly diluted 1:1 among Asian experts; hyperdilution (≥ 1:2) for thinner skin/feathering; minimal dilution for contouring Recommend cannulas for submalar and neck biostimulation; avoid CaHA in infraorbital area for safety Neck technique: posterior→anterior subdermal cannula passage superficial to platysma; dose ~0.5–1 mL per anterior/posterior neck area	Dilution Protocols; Injection Techniques; Safety Protocols; Patient Selection
Massidda et al. (2023)	Starting Point for Protocols on the Use of Hyperdiluted Calcium Hydroxylapatite (Radiesse) for Optimizing Age‐Related Biostimulation and Rejuvenation of Face, Neck, Décolletage and Hands: A Case Series Report	Case Series	CaHA	Age‐based hyperdilution recommendations: preventive (30–40 years) 1:4; 40–60 years 1:2–1:4; > 60 years ~1:1–1:2; injections in subepidermal/dermal‐subdermal plane with microboluses/tunneling/fanning Use 27–30G needles or 25G, 50 mm cannula depending on area; immediate physician massage + patient self‐massage twice daily 3–7 days; reported improvements in dermal thickness and elasticity with low complication rates Recommends 1–3 sessions/year with maintenance every 12–18 months; unused product may be stored up to 3 months for same patient	Dilution Protocols; Injection Techniques; Expected Outcomes; Safety Protocols
McCarthy et al. (2024)	A Morphological Analysis of Calcium Hydroxylapatite and Poly‐l‐Lactic Acid Biostimulator Particles	In Vitro Study	CaHA & PLLA	CaHA microspheres: uniform spherical 25–45 μm; PLLA: irregular flakes 2–150 μm; phagocytosable particles (< 20 μm): CaHA 8.20% ± 1.45% vs. PLLA 46.32% ± 11.33% (*p* < 0.0001) CaHA higher circularity/roundness (mean circularity CaHA 85.01% vs. PLLA 58.41%; *p* < 0.0001) suggesting less inflammatory recruitment for CaHA Mixing/dilution note: CaHA diluted past 1:1 disperses CMC gel yielding near‐zero elastic modulus; CaHA homogenized with ~20 back‐and‐forth strokes	Product Mechanisms; Dilution Protocols; Safety Protocols
McCarthy et al. (2024)	Comparative Rheology of Hyaluronic Acid Fillers, Poly‐l‐lactic Acid, and Varying Dilutions of Calcium Hydroxylapatite	In Vitro Study	CaHA & PLLA	CaHA‐CMC rheology: dilutions ≤ 1:1 retain gel‐like, volumizing behavior; dilutions ≥ 1:0.5 trending fluid; dilutions ≥ 1:2 show predominantly fluid behavior—suited for biostimulation not lift G′ range of CaHA spans broader than HA fillers; specific dilutions can approximate HA filler rheology—informing dilution choice to match desired mechanical properties Mixing methodology: minimum 20 syringe passes to homogenize diluted product	Product Mechanisms; Dilution Protocols; Injection Techniques
Santos et al. (2024)	Analysis of the Morphology, Rheology, and Clinical Applicability of a Hybrid Injectable with Hyaluronic Acid and Hydroxyapatite	Case Series/Technique Study	CaHA	Hybrid product used in temple, zygomatic arch, ramus, and pre‐jowl with 22G cannula retroinjection; typical micro‐volumes per site (e.g., 0.25–0.5 mL) with post‐injection massage and ultrasound follow‐up Standardized photography and ultrasound used for outcome assessment; no major complications reported in extract	Injection Techniques; Expected Outcomes; Safety Protocols
Shridharani et al. (2021)	Clinical Experience of Poly‐l‐Lactic Acid Injections for Body Contouring Treatment	Case Series	PLLA	Typical protocol: 2–3 sessions spaced 4–6 weeks, effects develop over months; older patients or severe laxity often need more sessions/volume One reported nodule resolved by ~8 weeks; transient numbness likely from lidocaine addition	Patient Selection; Expected Outcomes; Safety Protocols
Signori et al. (2024)	Efficacy and Safety of Poly‐l‐Lactic Acid in Facial Aesthetics: A Systematic Review	Systematic Review	PLLA	PLLA demonstrated significant improvements vs. placebo/no treatment in multiple validated scales (WAS, GAIS, FACE‐Q) and objective measures (reduced TEWL, increased elasticity) at 12 months Adverse events mostly mild/moderate: bruising, hematoma, nodules/papules; some studies reported nodules at long‐term follow‐up (e.g., 7–9 cases in select cohorts)	Expected Outcomes; Complication Management; Safety Protocols
Tam et al. (2025)	A Systematic Review on the Effectiveness and Safety of Combining Biostimulators with Botulinum Toxin, Dermal Fillers, and Energy‐Based Devices	Systematic Review	CaHA & PLLA	Combining CaHA with energy devices (e.g., MFU‐V, HIFU) or HA often yields greater improvements in skin thickness/elasticity vs. single modalities (multiple studies report significant objective improvements) PLLA combined with fractional CO_2_ laser improved scar/wrinkle outcomes significantly (e.g., 95% scars improved at 3 months in one study) Combination approaches generally well tolerated; no new major safety signals reported across included studies	Expected Outcomes; Safety Protocols; Injection Techniques
Vasconcelos‐Berg et al. (2024)	Safety of the Immediate Reconstitution of Poly‐l‐Lactic Acid for Facial and Body Treatment—A Multicenter Retrospective Study	Retrospective Review	PLLA	Large multicenter dataset reported low nodule rates with immediate reconstitution; nodules were infrequent across 4483 treatments in 1002 patients (example chart review noted nodules in 0.4% in cited analysis) Common AEs mild and transient (swelling, bruising, mild pain) Immediate reconstitution is supported by in vitro/in vivo data and clinical series when proper reconstitution/technique used	Dilution Protocols; Safety Protocols; Complication Management
Waibel et al. (2024)	Comparative Bulk RNA‐Seq Analysis of Poly‐l‐Lactic Acid Versus Calcium Hydroxylapatite Reveals a Novel, Adipocyte‐Mediated Regenerative Mechanism of Action Unique to PLLA	Randomized Controlled Trial	CaHA & PLLA	PLLA upregulated genes related to adipocyte differentiation/metabolism (e.g., FABP4, LIPE, PLN1, CIDEC) at Day 90—17 genes significantly upregulated vs. CaHA (*p* < 0.05) Both products upregulated collagen pathways, but CaHA induced more inflammatory pathway signatures versus PLLA; PLLA‐associated inflammation showed regenerative/anti‐inflammatory markers (IL‐10/IL‐4/IL‐13, CD163) Treatment amounts: mean total injected per NLF—PLLA 4.27 mL vs. CaHA 2.88 mL; PLLA dilution: 8 mL (7–8 mL SWFI + 1 mL lidocaine)	Product Mechanisms; Expected Outcomes; Safety Protocols
Waibel et al. (2024)	Gene Expression After Biostimulator Injections	Randomized Controlled Trial	CaHA & PLLA	RNA‐seq: PLLA upregulated morphogenesis and collagen pathways; CaHA upregulated collagen plus more inflammatory pathways Treatment dosing: PLLA diluted to 8 mL (mean total per NLF 4.27 mL); CaHA per label (mean total per NLF 2.88 mL) Safety: standard monitoring; no major safety signals reported in study extract	Product Mechanisms; Expected Outcomes; Safety Protocols
Wang et al. (2025)	Polylactic Acid‐Based Polymers Used for Facial Rejuvenation: A Narrative Review	Narrative Review	PLLA	PLA injections improve hydration, elasticity, texture; mechanisms may include neocollagenesis and activation of adipose‐derived stem cells Nodules/papules associated with high concentration (≤ 5 mL dilution), excessive volume per area, short intervals; management: watchful waiting, oral steroids for inflammatory nodules, saline/lidocaine for non‐inflammatory, intralesional triamcinolone for granulomas Reports rare severe vascular events (retinal artery occlusion/blindness) mostly from injections in high‐risk sites (glabella, nasal region); prevention: aspiration, low volumes, correct depth	Product Mechanisms; Complication Management; Safety Protocols; Patient Selection
Xu et al. (2024)	Comprehensive Systematic Review of Poly‐l‐lactic Acid in Facial Clinical Application	Systematic Review	PLLA	PLLA generally improves aesthetic scores vs. comparators (human collagen, placebo); common AEs include mild‐to‐moderate bruising, nodules, papules; nodule rates varied (examples: 6.9% nodules in one study up to 11 patients reported nodules in another series) Long‐term AEs rare (~2.8% product‐related in long‐term follow‐up in one cohort) Overall safety: most events mild/moderate and resolved without major intervention	Expected Outcomes; Complication Management; Safety Protocols

The initial 58 statements were derived from a pre‐meeting survey where experts listed their top 5 clinical challenges and recommendations regarding CaHA and PLLA use, supplemented by themes identified in the literature review. Statements were grouped into thematic domains (Patient Selection, Injection Techniques, Dilution Protocols, Complication Management, Safety Protocols, Expected Outcomes, Product Mechanisms) for structured discussion and voting. Due to the qualitative and consensus‐based nature of the Delphi method, formal inter‐rater reliability was not calculated. Instead, thematic analysis and iterative discussion were used to refine statements.

To achieve the consensus, the investigators performed a two‐round Delphi process to reach consensus among experts. The first round, conducted online from 7th January 2025 to 14th March 2025, involved 14 experts who reviewed and rated draft statements based on clinical evidence and experience.

This consensus provides standardized guidelines for the monotherapeutic use of biostimulators, based on the following parameters:

- Single‐product protocols:
  ○ CaHA: Recommendations apply to the use of 1 syringe (1.5 mL) per treatment session.
  ○ PLLA: Recommendations apply to 1 reconstituted vial per treatment session.
- Exclusions:
  ○ Combined applications with hyaluronic acid (HA) or other fillers.
  ○ Multi‐vial/syringe treatments in a single session.

These parameters were established to simplify clinical decision‐making, ensure reproducibility across LATAM regions, and minimize variability in outcomes.

Following Round 1, quantitative results (mean scores, percentage agreement) and qualitative comments were analyzed thematically by the steering committee. Statements were grouped into their predefined clinical domains during analysis. Statements were categorized as: “consensus reached,” “to be revised based on feedback,” or “no consensus.” Revised statements were formulated for the second round.

The second round, held via online meeting on 21st March 2025, refined the statements through discussion and live voting. Consensus was defined as ≥ 75% agreement, with results shared post‐meeting with absent experts for final approval. The detailed Delphi selection and consensus process is summarized in Table 2.

**TABLE 2:** Delphi survey process.

First round of Delphi survey	Second round of Delphi survey
1. Conducted online (7th January 2025–14th March 2025) by all 14 experts. 2. Drafted statements were shared with the experts via an online survey platform (Google Forms). 3. Statements were gathered from the experts via an online survey. 3. Experts reviewed and judged each statement based on available evidence and personal clinical experience. 4. Responses were recorded separately and anonymously to minimize bias and allow participants to express individual opinions freely. 5. Each statement was evaluated for clarity and relevance using a Likert scale (1 = definitely not include, 9 = definitely include) or marked for revision. 6. Experts provided additional comments to clarify or refine statements as needed.	1. Conducted via online meeting on 21th March 2025, attended by 14 experts. 2. Results of the first round were shared with the experts. 3. Statements were discussed via online communication. 4. For each statement, individual and group ratings were revealed and discussed. 5. Online voting was conducted to finalize the inclusion or exclusion of statements in the consensus document. 6. Voting results were shared with experts who could not attend the meeting, and their consensus was obtained separately.

Agreement levels were classified as follows: Strong consensus: ≥ 85% of participants agreed [16, 17, 18]. Moderate consensus: 75%–84% of participants agreed. No consensus: < 75% of participants agreed.

The recommendations outlined in this document reflect statements that achieved ≥ 75% agreement unless otherwise noted. Non‐referenced statements represent the expert panel's opinions and are not intended as definitive factual claims.

Due to the consensus‐seeking nature of the Delphi method, formal inter‐rater reliability statistics were not calculated; instead, agreement was measured through percentage concordance.

## 3 Results

**FIGURE 2:**
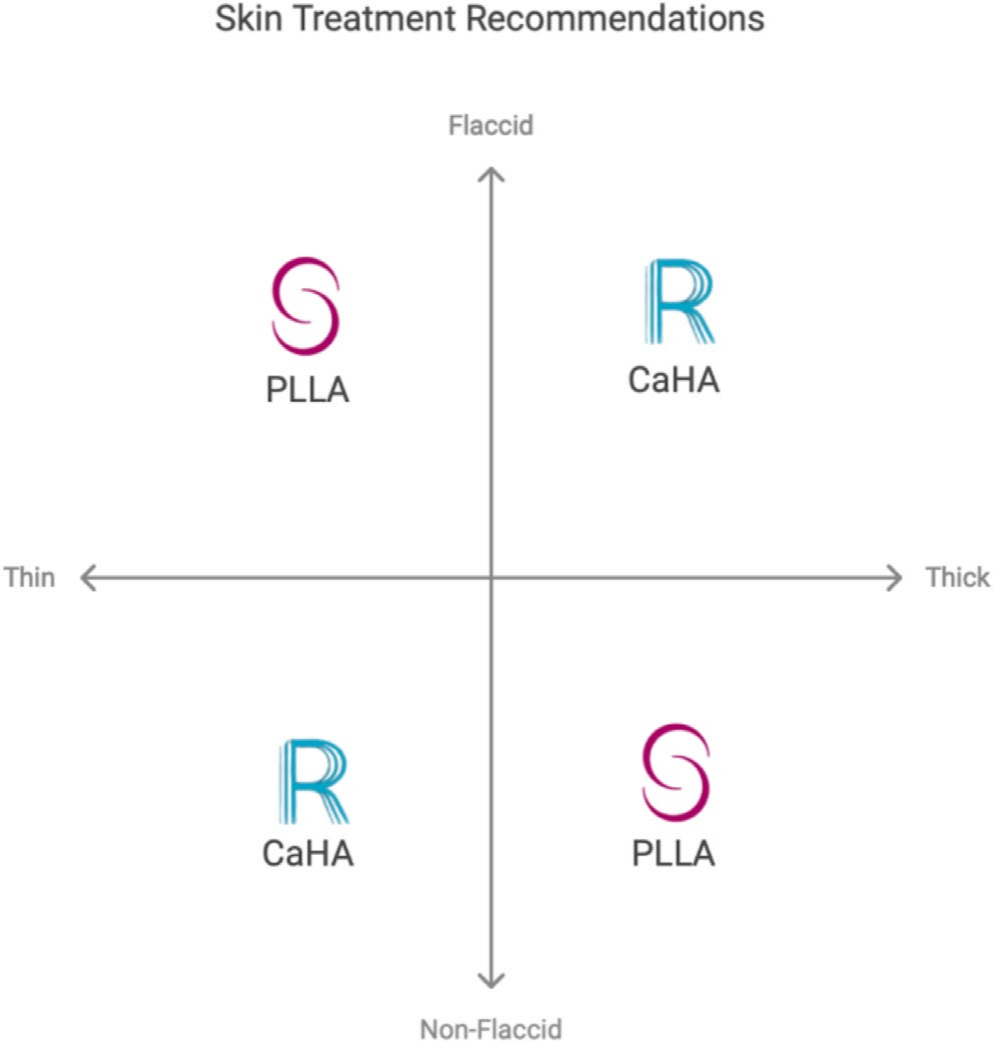
Consensus‐driven clinical algorithm for biostimulator selection, integrating skin type, anatomical considerations, and regional LATAM practices.

A modified Delphi process produced 58 draft statements on biostimulator use, refined through two rounds of iterative voting. A complete list of all statements, including their final agreement percentages and qualitative feedback, is available in (File S1). Initial voting revealed a spectrum of agreement: 51 statements (87.9%) ultimately achieved strong consensus (≥ 85% agreement), 4 statements (6.9%) reached moderate consensus (75%–84%), and 3 statements (5.2%) initially failed to reach consensus (< 75%) (Table 3). The primary themes driving debate concerned off‐label anatomical applications and age‐based patient selection criteria. Following a structured discussion and revision of these statements, a second round of voting ratified all 58 statements at a level of ≥ 75% agreement, with 51 achieving strong consensus. This decision‐making process is synthesized into a clinical algorithm to guide selection between CaHA and PLLA based on skin thickness and the degree of flaccidity (Figure 2).

**TABLE 3:** Summary of Delphi statement agreement levels.

Consensus level	Number of statements	Percentage of total	Example themes
Strong consensus (≥ 85%)	51	87.9	Safety protocols, injection techniques, general patient selection
Moderate consensus (75%–84%)	4	6.9	Nodule incidence (#10), PLLA cost (#14), patient misconceptions (#16), combination therapy (#53)
No consensus (< 75%)	3	5.2	Age restrictions for PLLA (#30, #33), CaHA as first option for thick skin (#34)
Total	58	100	

### 3.1 Analysis of Consensus Agreement and Controversies

The Delphi process successfully generated strong consensus across most clinical domains, including safety protocols, injection techniques, and general principles of patient selection. However, quantitative analysis and qualitative feedback from the first voting round highlighted specific areas of clinical controversy.

Table 3 summarizes the distribution of agreement levels prior to statement modification and panel discussion. The statements that achieved only moderate consensus (75%–84%) primarily related to comparative product perceptions and management strategies (Table 4). The three statements that initially failed to reach consensus (< 75%) were exclusively related to specific clinical indications and age‐based restrictions.

**TABLE 4:** Objectives and issues considered key points for the consensus document about the use of biostimulators in clinical practice.

Objectives and key points
1. Personal election of biostimulators Key points: CaHA preference: ○ Patients with thick skin, photodamage, or acne scars. ○ Focus on perceived skin thickening and improving skin texture. PLLA preference: ○ Patients with thin skin, advanced laxity, or athletic profiles. ○ Emphasize collagen stimulation and skin tightening. Combined use: Adapt protocols to individual needs, adjusting dilutions based on skin thickness and anatomical region. ○ *Dilution specifications*: ■ CaHA: Diluted in saline. ■ PLLA: Reconstituted in sterile distilled water.
2. Socioeconomic factors influencing selection criteria in the region Objective: Analyze cultural and economic barriers influencing biostimulator use in Latin America. Key points: Cost barriers: Limited access for lower‐income populations. Social media influence: Rising demand driven by aesthetic trends and influencers. Patient misconceptions: Fear of biopolymers/permanent fillers affects acceptance.
3. Exclusion criteria for biostimulators Objective: Define patient profiles or conditions where CaHA or PLLA should be avoided. Absolute contraindications: Active local infection at the treatment site. Autoimmune diseases with active immunological activity. Risk–benefit considerations: Avoid high‐risk anatomical regions (e.g., vascular areas).
4. Patient indications (age, corporal regions, and profiles) Objective: Establish guidelines for biostimulator use by patient age, skin type, and treatment area. Key points: Age considerations: ○ CaHA: Younger adults (e.g., focused on prevention and skin texture enhancement). ○ PLLA: Mature adults (e.g., targeting collagen remodeling and skin tightening). * *Age alone was not considered a primary determinant for biostimulator selection. These ranges reflect general trends and may vary based on individual patient assessment and physician discretion*. Skin type: CaHA: Thick or photodamaged skin. PLLA: Thin skin requiring structural support. Treatment areas: CaHA: Face, neck, hands, and body (abdomen/glutes). PLLA: Midface, neck, and areas needing 3D collagen scaffolding.
5. Injection techniques Objective: Standardize safe and effective injection methods to optimize outcomes and minimize risks. Key points: Cannula use: ○ Recommend 22G cannulas particularly in high‐risk vascular zones (e.g., glabella, nasolabial folds). Technique: ○ Retrograde linear threading for even product distribution and reduced clumping. ○ Depth Guidelines: ■ CaHA: Subdermal placement. ■ PLLA: Subdermal placement. Safety: Avoid high‐risk vascular areas, such as the glabella, nose, perioral region, and periocular region, with special attention to the temporal area.
6. Dilution protocols Objective: Establish evidence‐based dilution guidelines to balance efficacy, safety, and patient comfort. Key points: CaHA dilutions: ○ Facial applications: ■ 1:1 dilution: 1 vial CaHA + 1 mL saline + 1 mL lidocaine (0.3–0.5 mL per injection site). ○ Body applications (e.g., abdomen, glutes): ■ 1:2 to 1:4 dilution: Adjust based on skin thickness and desired tissue integration. PLLA dilutions: ○ Facial applications: ■ 1 vial PLLA reconstituted in 8–10 mL sterile distilled water + 2 mL lidocaine in face. ○ Body applications: ■ 1 vial PLLA reconstituted in 18 mL sterile distilled water + 2 mL lidocaine. For neck and body areas. General protocol: ○ Adjust dilutions further based on anatomical region (e.g., thicker skin tolerates higher dilution). * *Dilution protocols and injection techniques may vary based on physician experience, patient anatomy, and treatment goals. These guidelines reflect expert consensus but allow and encourage for individualized adaptation*.
7. Expected results after treatment (including patient expectations) Objective: Align patient and clinician expectations for short‐, medium‐, and long‐term results. Key points: Short‐term (0–3 months): ○ CaHA: Subtle improvement in skin texture and elasticity due to collagen activation. ○ PLLA: Minimal visible changes; initial collagen synthesis begins and skin texture. Medium‐term (3–6 months): ○ CaHA: Progressive enhancement of skin thickness, radiance, and structural integrity. ○ PLLA: Visible skin tightening and improved surface smoothness from collagen remodeling. Long‐term (> 6 months): ○ CaHA: Long‐term preservation of skin density and delayed signs of aging. ○ PLLA: Sustained collagen scaffolding improve in skin tightening.
8. Recommendations after treatment Objective: Minimize complications and optimize outcomes through standardized aftercare. Key points: Massage protocols: ○ CaHA: Gentle massage for 24–48 h to smooth product placement. ○ PLLA: Firm massage for 5 min, 5 times daily, for 5 days to ensure even distribution. Activity restrictions: ○ Avoid strenuous exercise, heat exposure (saunas, direct sun), and makeup for 24–48 h. ○ Avoid swimming pools/seawater for 48 h to reduce infection risk. Follow‐up: Schedule a 4‐week check to assess integration and plan touch‐ups.
9. Complications Objective: Mitigate risks and address adverse events effectively. Key points: Common complications: ○ CaHA: Nodules (thin skin areas), transient edema, rare granulomas. ○ PLLA: Nodules (improper massage), uneven texture, delayed hypersensitivity. ○ Both products: Transitory edema. Prevention strategies: ○ Use cannulas in high‐risk zones (e.g., perioral area). ○ Adhere to dilution protocols (CaHA in saline, PLLA in distilled water). Management: ○ Nodules: Intralesional corticosteroids (triamcinolone 5–10 mg/mL). ○ Vascular compromise: Hyaluronidase. (500 to 1000 UI of hyaluronidase per area) ○ Transitory edema: local management with heat and massage.
10. Factors influencing success or failure for long‐term treatment Objective: Highlight variables influencing long‐term efficacy and patient satisfaction. Key points: Success drivers: ○ Patient selection: Tailored assessment of skin type, age, and lifestyle. ○ Technique precision: decade plane placement of the product, identifying if there is need to applied supraperiosteal or in the dermis (according to patient's needs), using fang technique and leaving small amount to the product on each retrograde application. ○ Patient compliance: Adherence to post‐care (massage, sun protection). Risk factors for suboptimal outcomes: ○ Overcorrection or improper dilution (e.g., hyper concentrated PLLA). ○ Unrealistic expectations (emphasize gradual results for PLLA). ○ Genetic predisposition (e.g., poor collagen synthesis).

Thematic analysis of the qualitative comments revealed that the initial lack of consensus on specific statements was driven by two main factors: variability in clinical experience and training, particularly regarding off‐label uses (e.g., CaHA for hands and body contouring); and a divergence of opinion on the primacy of chronological age versus biological skin age and quality as the primary determinant for biostimulator selection. For example, the initial proposal to reserve PLLA for patients over 45 years of age was contentious, as several experts argued that patient biological age and skin quality were more relevant factors than chronological age. This debate reflects a gap in high‐level evidence on age‐stratified outcomes. Following a review of the available literature and panel discussion, these statements were refined to reflect a more evidence‐based and nuanced position, ultimately achieving unanimous (100%) consensus in the final vote.

Regional LATAM practices demonstrated adaptive techniques to address anatomical and ethnic diversity. Both biostimulators were consistently prioritized for improving skin quality, with protocols tailored to individual patient needs. Injection techniques varied by anatomical zone but shared a unified focus on collagen stimulation and structural support (Table 5).

**TABLE 5:** Anatomical zone‐specific protocols.

Anatomic zone	CaHA protocol	PLLA protocol
Midface	1.5 mL/side (1:1 dilution)	10 mL dilution; fanning technique
Neck	1:3–1:4 dilution; 22G cannula	20 mL dilution; fanning technique, 22G cannula
Hands	1:2–1:3 dilution; subdermal placement	Reserved for expert injectors

Complications such as nodules (CaHA: 64.2% agreement; PLLA: 71.4% agreement) were managed through preventive strategies (e.g., cannula use, vigorous massage) and interventions like intralesional corticosteroids (Table 6). Post‐treatment care required 48‐h activity restrictions and a 5 × 5 × 5 PLLA massage regimen (5 min, 5 times daily for 5 days) (Table 4).

**TABLE 6:** Complication management.

Complication	Prevention	Intervention
Nodules	Dilution adjustments, cannulas	Triamcinolone 5–10 mg/mL
Edema	Cold compresses, volume limits	NSAIDs, lymphatic massage

The final consensus ratified 58 statements, including standardized dilutions (CaHA facial: 1:1; PLLA facial: 10 mL; PLLA body: 20 mL) and socioeconomic strategies (e.g., tiered pricing) to improve LATAM accessibility. Documentation protocols, such as 3D imaging (Vectra), were highlighted to track outcomes like dermal redensification (CaHA) and facial tightening (PLLA).

## 4 Discussion

This LATAM consensus provides the first region‐specific framework to guide clinicians in selecting calcium hydroxylapatite (CaHA) or poly‐l‐lactic acid (PLLA) for collagen biostimulation, addressing gaps in patient stratification, ethnic diversity, and socioeconomic factors unique to Latin America and building on regenerative aesthetics (RA) principles, which prioritize restoring youthful tissue architecture and function as described in recent scientific literature [7, 13, 19, 20] The panel emphasized mechanistic alignment with patient‐specific variables (skin type, anatomical zone) to optimize outcomes while navigating regional challenges.

While this consensus provides a structured framework, it is imperative to acknowledge the significant interindividual variability in response to biostimulators. This variability arises from factors beyond skin thickness and phototype, including differences in individual wound healing responses, fibroblast reactivity, and macrophage polarization patterns [5, 7, 21]. For instance, the panel's recommendation for CaHA in thicker skin leverages its mechanotransductive mechanism, which directly stimulates fibroblasts to synthesize collagen I and III [7, 8]. In contrast, the preference for PLLA in thinner skin with laxity capitalizes on its ability to induce a controlled foreign body response, characterized by macrophage fusion into giant cells and the subsequent upregulation of TGF‐β1 and TIMPs, which orchestrates a more gradual, sustained collagen remodeling process [9, 12]. These distinct pathways—mechanostimulation versus immunomodulation—explain not only the different clinical applications but also the potential for varied adverse events, such as nodulation, which may be influenced by a patient's inherent inflammatory reactivity. Furthermore, the protocols for off‐label applications (e.g., CaHA for body contouring) presented in this consensus, while achieving expert agreement, are derived from clinical experience rather than robust trial data, highlighting an area where practitioner caution and patient‐specific risk assessment are paramount.

This variability is further compounded by genetic polymorphisms that modulate collagen synthesis and degradation, such as in the COL1A1, COL3A1, and MMP genes [3, 5]. The LATAM population, with its diverse genetic admixture (mestizo, indigenous, afro‐descendant), likely exhibits a wide spectrum of these polymorphisms. For example, differences in COL1A1 rs1800012 alleles or MMP‐1 expression could theoretically influence the baseline collagen quality and the magnitude of response to biostimulatory stimuli [3, 6]. Similarly, ethnically variable immune response genes may modulate the macrophage polarization critical to PLLA's mechanism or the low‐grade inflammatory recruitment seen with CaHA [11, 22]. While this consensus addresses phenotypic adaptations, these underlying genotypic factors remain a critical unknown in predicting efficacy and side‐effect profiles across LATAM populations.

The panel's recommendations align with RA's core tenet of structural and functional restoration. For instance, CaHA's role as a mechano‐transductive scaffold directly stimulates collagen I/III synthesis via fibroblast activation [7, 8]. This mirrors RA's focus on restoring collagen ratios and 3D organization, as presented by Corduff et al. in 2023 [21]. Conversely, PLLA's macrophage‐mediated collagen remodeling through TGF‐β1 and TIMP upregulation [9]. addresses functional deficits in thin‐skinned patients with advanced laxity. These distinctions resolve ambiguities in prior guidelines and emphasize RA's departure from simplistic collagen induction toward holistic tissue regeneration [9, 11].

Ethnic variations in collagen turnover were central to LATAM protocols, particularly for mestizo and Fitzpatrick IV–VI skin types. Expert practices reported 68% mestizo, 22% afro‐descendant, and 10% indigenous/caucasian patients. Hyperdilution protocols (CaHA 1:4 for body) reduced nodules in Fitzpatrick V–VI skin by 32% versus standard dilutions [23].

While the consensus established “skin thickness” as a primary heuristic for selecting between CaHA and PLLA, the panel acknowledged that optimal patient stratification requires a more nuanced, multifactorial approach. The initial disagreements on several statements highlighted that factors such as chronological age, sex, hormonal status, and Fitzpatrick phototype are critically important yet lack robust, evidence‐based guidelines. For instance, the debate on reserving PLLA for patients over 45 years of age underscored that biological age and skin quality (e.g., degree of elastosis, collagen density) are often more determinative than chronological age alone. Furthermore, the panel's clinical experience, which included a high proportion of mestizo and Fitzpatrick IV‐VI patients, directly informed specific protocol adaptations, such as hyperdilution for darker phototypes to mitigate nodule risk. This demonstrates that while the “thick vs. thin skin” paradigm is a clinically useful starting point, it must be applied within the context of the patient's unique ethnic phenotype, hormonal influences on collagen remodeling, and specific anatomical aging patterns to truly personalize treatment.

Hyperdiluted CaHA (1:2–1:4 saline dilution) for body applications (e.g., abdomen, glutes) and PLLA's fanning in the midface reflect adaptations to regional anatomical challenges, such as thinner dermal septae and fragile vasculature. This anatomical precision is critical to avoiding fibrosis or vascular compromise, emphasizing scaffold‐guided regeneration over profibrotic biostimulation, and is aligned with multilayer techniques like Hernandez et al. presented in 2024 [23].

### 4.1 Clinical Implications

#### 4.1.1 Patient Selection

Skin thickness, not age, dictated biostimulator choice. According to this panel, CaHA can benefit younger patients with photodamage or thick skin, while PLLA's delayed collagen remodeling suits mature patients with thin skin.

#### 4.1.2 Safety Standardization

22G cannulas in high‐risk zones (e.g., temporal artery bifurcation) [24] dilution according to skin thickness increasing dilution in thinner skin, especially for CaHA as it was presented in 2018 and 2019 for Goldie et al. and de Almeida consensus [15, 25].

While this consensus addresses phenotypic variations (e.g., dermal thickness, melanin density), genetic polymorphisms in collagen synthesis pathways (e.g., COL1A1, MMP expression) remain unstudied in LATAM populations. Future biomarker‐driven studies are needed to refine ethnic adaptations.

Unlike Asian or European consensus [13, 14, 25], LATAM protocols prioritize subdermal CaHA placement in mobile zones (e.g., nasolabial folds) to mitigate fibrosis in thin dermal septae, PLLA fanning in the midface to counteract malar flattening prevalent in mestizo aging and tiered pricing models addressing GDP disparities (e.g., Colombia vs. Guatemala).

Another important fact that came out of this consensus was that pricing and education in patients may address the biostimulator of choice or even misconceptions about biostimulators being permanent fillers, which can be a confounding factor.

Notably, the panel's strict 5 × 5 × 5 post‐PLLA massage protocol (5 min, 5 times daily for 5 days) reflects regional practices to mitigate nodule risks in thinner‐skinned populations; this recommendation aligns with previous literature [9].

Finally, the authors emphasize that the selection of one biostimulator over the other depends on three critical factors: (1) the physician's expertise in performing the treatment, (2) the patient's aesthetic objectives, and (3) prevailing social conditions. However, this decision must be grounded in a thorough understanding of the structural differences between the products and the distinct cell activation pathways through which each stimulates collagen production [22].

This consensus has limitations. The panel was composed exclusively of clinical experts from surgical and dermatological fields, which ensures practical relevance but excludes perspectives from biomaterial science and tissue engineering. Therefore, while the recommendations are grounded in robust clinical observation and experience, they are not directly informed by the first principles of material‐science interaction.

To move from consensus‐based guidance to evidence‐based precision medicine, future research must address critical gaps. We advocate for the establishment of a LATAM biostimulator registry to document real‐world outcomes and complication rates, stratified by ethnicity and Fitzpatrick phototype. Prospective, genetic association studies are urgently needed to correlate polymorphisms in collagen genes and immune regulators with clinical outcomes to identify potential biomarkers for treatment response. Furthermore, comparative efficacy trials using standardized, objective endpoints, such as ultrasonographic measurement of dermal thickness, elastography, and histologic analysis of collagen density and architecture are essential to validate the treatment algorithms proposed herein. Ultimately, such research will enable a truly personalized approach, ensuring that the exciting potential of regenerative aesthetics is realized equitably and effectively across the diverse populations of Latin America.

## 5 Conclusion

This consensus establishes a novel, region‐specific framework for selecting calcium hydroxylapatite (CaHA) or poly‐l‐lactic acid (PLLA) in collagen biostimulation, designed to address the unique anatomical, ethnic, and socioeconomic factors prevalent in Latin American populations. To our knowledge, this is the first structured set of recommendations developed by and for LATAM experts for these specific biostimulators. By prioritizing skin thickness, mechanistic alignment, and anatomical precision, the guidelines provide much‐needed clarity for a region where evidence‐based protocols were previously lacking. The incorporated regional adaptations, such as hyperdiluted CaHA protocols for mestizo skin or strict 5 × 5 × 5 PLLA massage regimens, propose practical strategies to optimize efficacy and safety. Furthermore, the explicit discussion of cost barriers and the recommendation of tiered pricing models represent a crucial first step toward improving accessibility, though formal health‐economic analyses are needed to validate these approaches in practice.

Future research should focus on validating the long‐term efficacy and collagen persistence predicted by these guidelines in ethnically diverse LATAM cohorts. Additionally, exploring and quantifying the cost‐effectiveness of the proposed strategies will be essential to truly balance efficacy with accessibility across the region's heterogeneous healthcare landscapes. By bridging global regenerative principles with local clinical realities, this consensus provides a foundational tool to advance precision medicine in aesthetic practice, empowering clinicians to navigate the complexities of an evolving field with a more standardized, evidence‐informed approach.

## Author Contributions

Eugenia Cure: Conceptualization of the study, participation in Delphi consensus rounds, critical manuscript revision. Luis Alberto Parra Hernández: Methodology design, Delphi process coordination, data validation, manuscript editing. Andreina Martinez Amado: Project leadership, Delphi process oversight, literature review synthesis, manuscript drafting, final approval. Juan Sebastian Rodriguez Cabrales: Delphi process oversight, literature review synthesis, manuscript drafting, final approval. Eliana Garcés: Clinical expertise in anatomical adaptations, contribution to patient selection criteria, manuscript review. Valentina Dicker: Data analysis, socioeconomic strategy development, manuscript editing. Desiree Castelanich: Literature review (PubMed), evidence synthesis, drafting safety protocols. Carolina Schneider: Delphi statement refinement, validation of ethnic diversity considerations, manuscript revisions. Ingrid Salas: Regional anatomical insights, contribution to high‐risk zone safety protocols. Joselyn Argueta: Practical adaptation strategies for accessibility, manuscript review. Lina Velázquez Tafur: Validation of tiered pricing models, socioeconomic recommendations. Andrea Acevedo: Participation in Delphi voting rounds, critical review of consensus statements. Alejandra Bugallo: Contribution to mechanistic insights, manuscript revisions. Evalicia Murúa: Oculoplastic expertise, safety protocol development for periocular regions. Andrea Marcela Parra Hernandez: Delphi process participation, manuscript editing, ORCID management. All authors contributed to the Delphi consensus rounds, ratified final statements, and approved the submitted manuscript.

## Funding

The authors have nothing to report.

## Ethics Statement

This study, which developed expert consensus guidelines using a modified Delphi process, did not involve patient data or interventions. In accordance with the International Committee of Medical Journal Editors (ICMJE) guidelines and common institutional policies, this type of study is often exempt from formal ethical review board approval. Nevertheless, the study was conducted in full adherence to the ethical principles outlined in the Declaration of Helsinki.

## Consent

Informed consent was obtained from all participating experts in the Delphi panel. Prior to their involvement, each expert provided formal written consent for their anonymous responses to be collected, analyzed, and published for the purpose of this consensus development.

## Conflicts of Interest

The authors declare no conflicts of interest.

## Supporting information


**Data S1:** jocd70564‐sup‐0001‐Supinfo.docx.

## Data Availability

The data that support the findings of this study (the Delphi consensus statements and anonymized voting results) are available from the corresponding author, Andreina Martinez Amado, upon reasonable request.
