# Supplementary material for: Optimizing Collagen Biostimulator Choice in LATAM: Expert Consensus on Patient Selection, Ethnic Skin Phenotypes, and Accessibility
Source: J Cosmet Dermatol. 2025 Nov 28;24(12):e70564. doi: 10.1111/jocd.70564 (PMC12663610; doi:10.1111/jocd.70564)
Supplement: Supplementary file 1 — Data S1: jocd70564‐sup‐0001‐Supinfo.docx. [file JOCD-24-e70564-s001.docx]

**Optimizing Collagen Biostimulator Choice in LATAM: Expert Consensus on Patient Selection, Ethnic Skin Phenotypes, and Accessibility**

Agreement levels were classified as follows:

Strong consensus: ≥85% of participants agreed.

Moderate consensus: 75–84% of participants agreed.

No consensus: <75% of participants agreed.

During our second round—a virtual meeting held in accordance with our methodological strategy—a strong consensus had not yet been achieved. We therefore asked the authors to share their comments and suggestions for modifying the statements. Following discussion, a virtual vote was conducted, resulting in a final consensus among the authors. The statements presented here reflect this final agreed version. We also include an explanation of why consensus was not initially reached. For each statement where strong consensus was lacking initially, both the initial and final agreement percentages are provided.

**Statements**

1. The choice between CaHA and PLLA depends primarily on the practitioner's subjective experience, not on standardized protocols.

Strong consensus 92.8%

1. Regional differences are due to variations in medical training, product availability, or the influence of local opinion leaders.

Strong consensus 85.7%

1. The lack of regional consensus leads to the selection of biostimulators being guided by individual experience rather than collective evidence.

Strong consensus 100%

1. Significant results are not expected in the short term with CaHA or PLLA; their effects are progressive.

Strong consensus 85.7%

1. In the medium term, both biostimulators improve skin firmness and elasticity.

Strong consensus 100%

1. In the long term, CaHA and PLLA promote dermal redensification and control of sagging.

Strong consensus 100%

1. The facial tightening effect is a key long-term result, especially with PLLA.

Strong consensus 85.7%

1. Increased skin thickness and hydration are consistent effects of CaHA and PLLA.

Strong consensus 92.8%

1. CaHA offers more immediate results in volume, while PLLA acts gradually in collagen stimulation.

Strong consensus 85.7%

1. Nodule formation is the most frequent complication associated with the use of CaHA and PLLA.

Moderate consensus 76.9%

1. CaHA has a higher incidence of nodules compared to PLLA.

Initial statement:

- Agree 61.5%
- Neutral 23%
- Disagree 15.3%

The disagreement arose because some experts contended that there is insufficient evidence to conclude that CaHA has a higher rate of complications compared to PLLA. However, when reviewing the available literature, we found a greater number of documented case reports involving nodule formation associated with CaHA relative to those reported for PLLA.

Final statement:

Strong consensus 100%

1. The formation of nodules is related to the application technique (e.g., superficial injection or excessive volume)

Strong consensus 92.8%

1. The high cost of biostimulators limits their access to a specific niche of patients in Latin America.

Strong consensus 92.8%

1. PLLA is perceived as more expensive than CaHA, which influences its selection.

Moderate consensus 78.5%

1. Social media and influencers have a significant impact on patients' decisions to request biostimulators.

Strong consensus 85.7%

1. The fear that biostimulators may behave like illegal biopolymers affects their acceptance among some patients.

Moderate consensus 78.5%

1. Advertising and local aesthetic trends drive the preference for specific brands of CaHA or PLLA.

Strong consensus 85.7%

1. Hyperdilution of CaHA and PLLA reduces the risk of complications such as nodules.

Strong consensus 85.7%

1. Physicians should receive specific training in facial anatomy and the rheological properties of biostimulators.

Strong consensus 92.8%

1. The selection of the biostimulator should be based on individual characteristics (e.g., skin type, degree of sagging, expectations).

Strong consensus 85.7%

1. High-quality products and regulatorily approved brands should be prioritized to ensure safety.

Moderate consensus 78.5%

1. Post-treatment massage is essential to distribute the product and prevent nodules, especially with PLLA.

Strong consensus 92.8%

1. The consensus should standardize safe application techniques (e.g., use of cannula vs. needle, anatomical planes).

Strong consensus 92.8%

1. Specific dilution protocols should be defined by anatomical zone (e.g., face 1:1, body 1:4).

Strong consensus 85.7%

1. The selection of CaHA or PLLA should be based on: skin type (Fitzpatrick), skin thickness, degree of sagging, and patient age.

Strong consensus 92.8%

1. Guidelines for the treatment of nodules should be established (e.g., massage, intralesional corticosteroids).

Strong consensus 85.7%

1. CaHA is the first option for facial contour definition (e.g., jawline, cheekbones).

Strong consensus 85.7%

1. PLLA is prioritized in patients with marked sagging or moderate to severe loss of ligament support.

Strong consensus 100%

1. The hands are a key area for the use of CaHA.

Initial statement:

- Agree 64.3%
- Neutral 35.7%

The lack of consensus arose because, given the variety of products and treatments they use, not all experts considered CaHA suitable for the hands, and some lacked experience in this specific area. Nevertheless, when referring to the available literature, we found clear indications supporting the use of this product for hand rejuvenation treatments.

Final statement:

Strong consensus 100%

1. PLLA is reserved for patients over 45 years of age with advanced sagging.

Initial statement:

- Agree 64.2%
- Neutral 21.4%
- Disagree 14.2%

The reason there was discussion regarding this statement is that some experts did not restrict the use of PLLA to patients over a certain age, nor exclusively to those with advanced sagging. When reviewing the literature, we found that CaHA generally has a broader application in younger patients, while PLLA was more commonly preferred as a long-term reparative mechanism.

Final statement:

Strong consensus 100%

1. There are no clinically valid reasons to avoid the use of CaHA or PLLA if standardized protocols are followed.

Strong consensus 85.7%

1. CaHA is preferred in young patients (approximately 25-45 years old) for prevention of aging and improvement of skin quality.

Strong consensus 92.8%

1. PLLA is more suitable for patients over 45 years of age with moderate to severe sagging.

Initial statement:

- Agree 57.1%
- Neutral 42.8%

The discussion occurred because experts believed that the use of PLLA should not be limited to older patients. However, after reviewing the literature and finding that this product is generally reserved for older patients with more advanced sagging, consensus was reached.

Final statement:

Strong consensus 100%

1. In patients with thick or photoaged skin (Fitzpatrick III–IV), CaHA is the first option.

Initial statement:

- Agree 71.4%
- Neutral 28.6%

The reason this statement was debated is that some experts did not necessarily consider CaHA to be the first-line treatment for patients with thick or photoaged skin. However, the literature indicates that while it may not always be the absolute first choice, it is indeed one of the preferred treatments for these skin types.

Final statement:

Strong consensus 100%

1. PLLA is preferred in thin skin or areas with low adipose tissue to avoid nodules.

Strong consensus 92.8%

1. Individualization of therapy is essential: there is no single protocol for all patients.

Strong consensus 92.8%

1. For facial applications, CaHA should be diluted in a 1:1 ratio (5 ml of Radiesse + 1.5 ml of diluent).

Strong consensus 76.9%

1. PLLA should be reconstituted with 8 to 10 ml of sterile water + 2 ml of lidocaine for facial use.

Strong consensus 85.7%

1. For body areas (abdomen, glutes), CaHA should be diluted 1:4 to optimize coverage and safety.

Strong consensus 85.7%

1. CaHA is the biostimulator of choice for the face (middle and lower third).

Moderate consensus 78.5%

1. PLLA should be reserved for the upper facial third (forehead, temples) due to its gradual collagen stimulation and natural results.

Strong consensus 92.8%

1. CaHA is not the first option for extensive body areas (e.g., abdomen) due to its low coverage and durability performance.

Initial statement:

- Agree 71.4%
- Neutral 21.4%
- Disagree 7.1%

The reason this statement was debated is that not all experts agreed with the assertion that CaHA should not be considered a first-line treatment. However, the literature supports its classification as a therapeutic option rather than a primary choice for these areas.

Final statement:

Strong consensus 100%

1. Areas with extremely thin skin (e.g., periorbital region) should be avoided for both CaHA and PLLA.

Strong consensus 100%

1. Flexibility in application (e.g., off-label use) depends on the physician’s experience, not on laboratory guidelines.

Strong consensus 92.8%

1. Ideally, sessions of CaHA and PLLA should be separated by at least 4 weeks to reduce risks and evaluate progressive results.

Initial statement:

- Agree 69.2%
- Neutral 30.8%

The discussion arose because, while experts agreed on the need for treatment follow-up, the timeframe could vary—often taking up to 6 weeks for either product to show visible results. Additionally, experts emphasized that complication management should be addressed as immediately as possible.

Final statement:

Strong consensus 100%

1. Intense physical exercise should be avoided for 24-48 hours post-treatment to reduce the risk of edema and product migration.

Strong consensus 84.6%

1. Exposure to heat (saunas, steam baths) should be avoided for 48 hours to prevent inflammation.

Strong consensus 100%

1. Makeup should not be applied during the first 24 hours to avoid contamination of the injection sites.

Strong consensus 92.3%

1. Contact with pool or sea water should be avoided for 48 hours to prevent infections.

Strong consensus 92.8%

1. The lack of alignment between expected and real results is a common cause of perceived failure.

Strong consensus 92.8%

1. The physician's clinical experience and anatomical knowledge are decisive in preventing complications.

Strong consensus 100%

1. If there is no visible improvement after 6 months, it is indicated to repeat the biostimulation or combine it with other therapies (e.g., radiofrequency, microneedling).

Strong consensus 92.8%

1. The combination of CaHA and PLLA can be synergistic, addressing both immediate volume loss and long-term collagen remodeling.

Moderate consensus 76.9%

1. Patient education on the progressive nature of results is crucial for managing expectations and ensuring satisfaction.

Strong consensus 100%

1. The properties of CaHA make it particularly suitable for structural support in areas like the mandibular border and chin.

Strong consensus 92.8%

1. Economic factors and product availability within the Latin America require a flexible approach to biostimulator selection without compromising safety and efficacy.

Strong consensus 100%

1. For practitioners new to biostimulators, starting with CaHA can be advantageous due to its more forgiving nature and immediate visual feedback during injection.

Strong consensus 84.6%

1. Patient follow-up beyond 12 months is recommended to fully assess the duration of effect and the need for maintenance sessions.

Strong consensus 100%
